# Triplet Acetone Generation by Pseudoperoxidase Activity of Myoglobin: Structural Damage and Quenching

**DOI:** 10.1002/bio.70618

**Published:** 2026-09-09

**Authors:** Thiago M. V. Gomes, Cassius V. Stevani, Kostas Pantopoulos, Luiz D. Ramos, Etelvino J. H. Bechara

**Affiliations:** ^1^ Departamento de Bioquímica, Instituto de Química Universidade de São Paulo São Paulo Brazil; ^2^ Lady Davis Institute for Medical Research, Jewish General Hospital and Department of Medicine McGill University Montreal Quebec Canada; ^3^ Departamento de Química Fundamental, Instituto de Química Universidade de São Paulo São Paulo Brazil; ^4^ Centro de Ciências Naturais e Humanas Universidade Federal do ABC Santo André São Paulo Brazil; ^5^ Instituto de Ciências Ambientais, Químicas e Farmacêuticas Universidade Federal do Estado de São Paulo Diadema São Paulo Brazil

**Keywords:** chemiexcitation, hemoprotein, isobutanal, myoglobin, triplet acetone

## Abstract

Triplet‐state carbonyl species can be generated in vivo through the oxidation of suitable substrates catalyzed by heme proteins and peroxynitrite, suggesting their involvement in metabolic processes and oxidative stress. In this work, we identified the formation of electronically excited triplet‐state acetone during the aerobic oxidation of isobutanal (IBAL) initiated by ferrimyoglobin (ferriMb). This system was compared with the well‐established IBAL/horseradish peroxidase (HRP) chemiluminescent reaction. Evidence for triplet acetone formation included Soret band spectral changes during the reaction, ultraweak light emission, and sodium 9,10‐dibromoanthracene‐2‐sulfonate (DBAS)‐promoted enhanced emission, as well as quenching by diene quenchers like sorbic acid and ethyl sorbate. Oxygen uptake measurements obeyed first‐order kinetics in oxygen concentration for the ferriMb system. Induced structural modification of ferriMb by IBAL was identified. Together, these findings suggest that ferriMb may drive the generation of harmful triplet species under carbonyl stress. Notably, rhabdomyolysis is reportedly known as an injurious condition in which free myoglobin is present in the bloodstream.

## Graphical abstract

Ferrimyoglobin pseudoperoxidase activity was demonstrated during isobutyraldehyde oxidation, yielding electronically excited triplet acetone and inducing structural damage. Sorbates and 9,10‐dibromoanthracene‐2‐sulfonate exhibited protective effects.

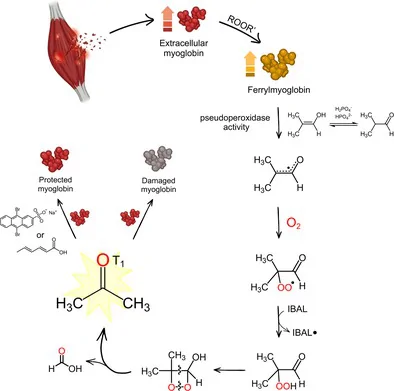

**Abbreviations**
DBA: 9,10‐dibromoanthracene
DBAS: 9,10‐dibromoanthracene‐2‐sulfonate anion
DTPA: diethylenetriaminepentaacetic acid
GC–MS: gas chromatography–mass spectrometry
Hb: hemoglobin
HEI: high‐energy intermediate
HEPES: 4‐(2‐hydroxyethyl)‐1‐piperazineethanesulfonic acid
HRP: horseradish peroxidase
IBAL: iso‐butanal
Mb: myoglobin
PUFAs: polyunsaturated fatty acids
SAH: subarachnoid hemorrhage
SDS‐PAGE: sodium dodecyl sulfate–polyacrylamide gel electrophoresis

## 1 Introduction

During the 1970s, the endogenous formation of excited species in non‐irradiated tissues was hypothesized to be physiologically relevant, then named “photochemistry in the dark” [1, 2, 3]. According to this model, and contrary to classical photochemistry, “photo”products arise not only from irradiation but also from electronic chemiexcitation. High‐energy intermediates of chemiluminescence (CL) reactions, such as 1,2‐dioxetanes, were proposed to undergo thermal cleavage yielding electronically excited carbonyl products via thermolysis. Predominantly triplet states are formed from alkyl and aryl dioxetanes, resulting in ultraweak light emission in aerated aqueous medium [4]. In turn, due to their long lifetimes and radical reactivity, triplet carbonyls can initiate biological one‐electron processes and transfer energy to adequate fluorophores [5].

**FIGURE 1:**
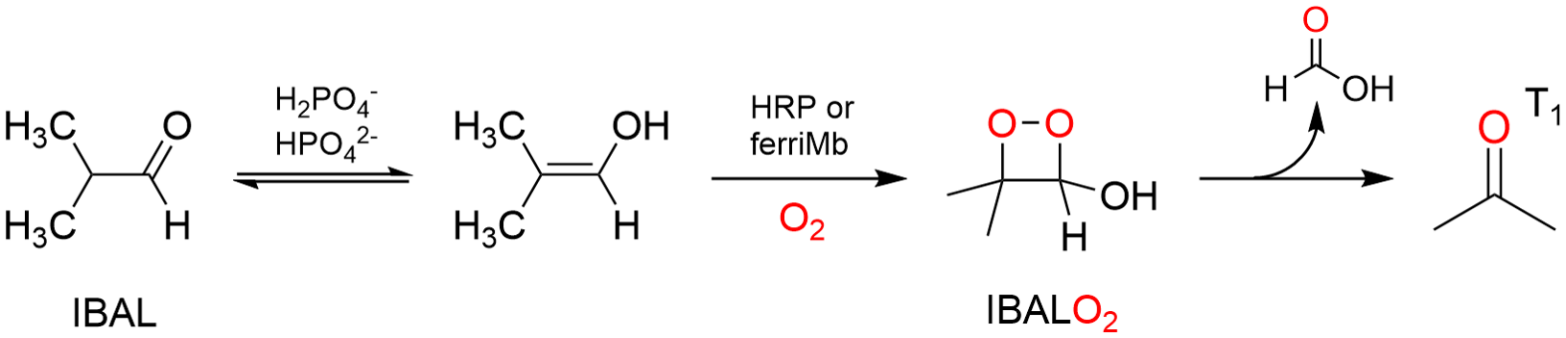
Proposed mechanism for enzymatic generation of triplet acetone through IBAL oxidation via a dioxetane as a high‐energy intermediate of the ultraweak CL.

Horseradish peroxidase (HRP)‐catalyzed oxidation of isobutanal (IBAL) in an aerated phosphate buffer at physiological pH represents a classic model enzymatic reaction that yields acetone at the triplet excited state via a high energy 1,2‐dioxetane intermediate (HEI) (Figure 1) [6, 7, 8]. The reaction is reported to proceed via previous phosphate‐catalyzed enolization of IBAL, followed by hydrogen abstraction from the enol by HRP intermediates I and II. The enoyl radical propagates the reaction while undergoing a competing addition of molecular oxygen. Cyclization of the resulting IBAL *α‐*hydroperoxide leads to the formation of a hydroxy dioxetane, whose thermal cleavage yields formic acid and acetone in the triplet excited state [6]. Importantly, triplet‐triplet energy transfer to ground state O_2_ (^3^Σ_g_^−^) yields reactive, electrophilic singlet oxygen (^1^Δ_g_) [9, 10, 11].

Reportedly, upon peroxidase catalysis, ketones like acetoacetate and 2‐methylacetoacetate as substrates yield excited methylglyoxal and biacetyl, respectively, showing maximal activity at pH 5.8 [12]. In turn, the enolate form of IBAL or 2‐phenylpropanal, sources of excited triplet acetone and acetophenone, respectively, presents higher activity at pH values above 7.0 [13, 14]. It is worth noting that phosphate and borate enable bifunctional catalysis, whereas Tris and acetate buffers do not. On the other hand, HRP in the presence of liposomes or micelles or even hematin alone was also previously documented to catalyze the aerobic oxidation of IBAL to excited acetone and formic acid [15, 16].

Myoglobin (Mb) is a hemoprotein representing approximately 2% of the muscle tissue protein. It is expressed in both skeletal and cardiac muscles and plays an essential role in oxygenation of these tissues [17]. However, it is vulnerable to oxidation to ferryl species (Mb‐Fe^IV^=O) in the presence of H_2_O_2_ and lipid peroxides, both in vitro [18, 19] or in vivo [20]; these species have been detected in a suspension of adult rat cardiac myocytes [21]. Ferrylhemoglobin (ferrylHb) has likewise been detected in red blood cells and in human blood [22, 23].

It is well established that ferrylmyoglobin (ferrylMb) exhibits pseudoperoxidase and pro‐oxidant activity [24, 25]. In the catalytic cycle of peroxidase enzymes, ferrylMb is present both as Compound I, characterized by the presence of Mb‐Fe^IV^=O and a porphyrin π‐cation radical, and Compound II, characterized by the presence of only Mb‐Fe^IV^=O [26]. FerrylMb activity can initiate the peroxidation of polyunsaturated fatty acids (L‐H) through peroxyl radicals intermediates (LOO•), whose annihilation produces singlet oxygen, the corresponding carbonyl compound and alcohol, according to the Russell mechanistic pathway [27]. The investigation of Mb pseudoperoxidase activity towards carbonyl substrates as a potential source of electronically excited triplet carbonyl species is of particular interest, as it may contribute to understanding the role of reactive carbonyls under conditions of elevated extracellular Mb levels, such as those observed in rhabdomyolysis.

Rhabdomyolysis, a condition resulting from the breakdown of skeletal muscle, results in the release of Mb and several intracellular muscle constituents into the bloodstream. It can be triggered by a variety of traumatic or non‐traumatic causes, including excessive physical exertion, substance abuse, and crush and blast injuries [28, 29, 30]. Subarachnoid hemorrhage (SAH), an acute neurovascular emergency, is characterized by blood leakage between the arachnoid membrane and the pia mater, most commonly due to the rupture of an intracranial aneurysm (approximately 80% of cases) [31]. Rhabdomyosarcoma is a rare and aggressive malignant tumor of striated muscle, primarily affecting children and young individuals [32]. Mb is a key biomarker in rhabdomyosarcomas, useful not only for diagnosis but also for differentiation from other soft tissue tumors, as well as for indicating different stages of tumor differentiation [33, 34, 35, 36]. In all these conditions, Mb and hemoglobin (Hb) reach abnormally high levels, either extracellularly or within tumor cells, thereby exposing these heme proteins to an oxidizing environment that favors the formation of ferrylMb and ferrylHb [37].

In this work, we demonstrate that ferrimyoglobin (ferriMb) triggers the oxidation of IBAL to electronically excited triplet acetone, generating ultra‐weak CL in phosphate buffer at physiological pH, and concomitantly inducing oxidative damage to the protein (Figure 1).

## 2 Experimental

### 2.1 Reagents and Solutions

Equine heart Mb, HRP type VI, sodium monobasic and sodium dibasic phosphate monohydrate, diethylenetriaminepentaacetic acid (DTPA), HEPES, potassium ferricyanide, 9,10‐dibromoanthracene (DBA), NaOH, chlorosulfonic acid, acetic acid, acetonitrile, sodium sorbate, and ethyl sorbate were purchased from Sigma‐Aldrich. Tris–HCl was obtained from ACS Científica and analytical grade 30% (v/v) hydrogen peroxide from Vetec. Isobutyraldehyde (Sigma‐Aldrich) was freshly distilled prior to use and dissolved in acetonitrile.

### 2.2 Preparation of 9,10‐Dibromoanthracene‐2‐sulfonate (DBAS)

Sodium DBAS was prepared following a modified procedure based on the method of Morley [38]. Glacial acetic acid (20.6 mL) was added to a round‐bottom flask, followed by dropwise addition of chlorosulfonic acid (7.9 mL), and the mixture heated to 50°C. Subsequently, DBA (4.0 g) was added, and the reaction was stirred at 50°C for 4 h. The resulting solution (28.5 mL) was transferred to an equal volume of hot water and brought to boiling. The hot mixture was vacuum‐filtered, and the retained solid washed with boiling water (2.0 L) until no fluorescence was detected under UV irradiation (365 nm). The pH was adjusted to 7.0, and solvent removed by rotary evaporation. The resulting solid was ground to a fine powder and washed with acetonitrile (0.90 L) to remove inorganic salts, followed by vacuum filtration. After complete solvent removal by rotary evaporation and lyophilization, 125 mg of product was obtained (approximately 3.13% yield). Product identification was made by HPLC, UV–Vis absorption, fluorescence, and NMR spectroscopy.

### 2.3 FerriMb Preparation

FerriMb was prepared by adding 10% (w/w) potassium ferricyanide to 10 mg of commercial Mb dissolved in 100 mM phosphate buffer, pH 7.4, containing 0.10 mM DTPA. After stirring for 30 min at room temperature, the mixture was desalted using a HiTrap desalting column connected to an ÄKTA Pure (Cytiva) chromatograph. Fractions were collected automatically, allowing separation of the protein from the salt‐containing fractions.

### 2.4 Spectrophotometric Studies

Spectrum changes of the Soret band during the IBAL aerobic oxidation catalyzed by ferriMb or HRP were recorded on a NanoPhotometer NP80 IMPLEN from 200 to 900 nm. Unless otherwise stated, the reactions were carried out in a 10 mm optical path quartz cuvette holding 100 mM phosphate buffer, pH 7.4, using 50 μM ferriMb with 10 μM H_2_O_2_ and 50 mM IBAL or 5 μM HRP, 0.5 μM H_2_O_2_, and 20 mM IBAL. The reactions were initiated with the addition of hemoprotein. For concentrated samples, the NP80 IMPLEN automatically reduces the optical path length to keep absorbance within the linear range (e.g., 1.0 absorbance units) to calculate the actual concentration.

### 2.5 Energy Transfer and Stern–Volmer Analysis

Ultraweak CL from IBAL/H_2_O_2_/ferriMb (HRP) reaction was monitored using a total photon counting tube luminometer (Berthold, Sirius). The effect of substrate, enzyme, buffer, and pH on light emission was assessed by varying the concentrations of IBAL (10–150 mM), ferriMb or HRP (5–50 μM), and phosphate (50–500 mM), pH (4.2–8.0), as well as buffer composition (100 mM phosphate, Tris or HEPES). Light enhancement by DBAS was measured both in the presence and absence of 1.0 mM sorbic acid or ethyl sorbate. Quenching rate constants (k_q_) were determined from the slope of Stern–Volmer plots, according to Equations (1) and (2), in which I_0_ and I are CL intensity in absence or presence of sorbic acid or ethyl sorbate (0.10–1.0 mM), respectively, [Q] is quencher concentration and τ is the lifetime of the excited donor species.

(1) $$I_{0}/I=1+K_{\mathrm{SV}}\;\left[Q\right]$$

(2) $$K_{\mathrm{SV}}=k_{q}\tau$$

### 2.6 Oxygen Uptake

Oxygen uptake was monitored on an Oxygraph‐O_2_ (Oroboros) equipped with a Clark‐type electrode. Oxygen concentrations were traced from the reaction mixture with 100 μM H_2_O_2_, 10 up to 100 mM IBAL, plus 10 μM HRP or ferriMb for 15 or 40 min, respectively.

### 2.7 SDS‐PAGE

SDS‐PAGE experiments were carried out on 15% acrylamide gel. Solutions containing 1.0 mg mL^−1^ (60 μM) ferriMb and 10 μM H_2_O_2_, in the presence or absence of 50 mM IBAL, 1.0 mM sorbic acid or ethyl sorbate, with and without 10 μM DBAS, were incubated at 37°C for 24 h and then subjected to vertical electrophoresis under 100 V for 90 min. Coomassie Blue was used for band staining. Gels were scanned using an Odyssey DLx (LI‐COR Biotech) imaging system. Image acquisition was performed with LI‐COR Acquisition Software, and densitometric analysis was carried out using the image processing and analysis software ImageJ.

### 2.8 Product Detection by GC–MS

A reaction mixture containing 100 mM IBAL, 500 μM ferriMb, and 5.0 mM H_2_O_2_ was incubated under pure oxygen exposure for 24 h at 25°C and 900 rpm. Acetone was detected using a gas chromatograph coupled to a mass spectrometer equipped with an electron impact ionization source and a quadrupole analyzer (Shimadzu GCMS‐QP2010 Ultra). A nonpolar Agilent DB‐5ms Ultra Inert capillary GC column ((5%‐phenyl)‐dimethylpolysiloxane stationary phase) with dimensions of 30 m length × 0.25 mm inner diameter (ID) × 0.25 μm film thickness was used. Helium was employed as the carrier gas at a total flow rate of 4.6 mL min^−1^ (column flow: 1.78 mL min^−1^) and a pressure of 14.5 psi. The oven temperature program started at 40°C and was held for 3 min, followed by a heating ramp of 3°C min^−1^ up to 100°C. A second ramp of 20°C min^−1^ was then applied up to 150°C, which was maintained for 4.5 min totaling 31 min of analysis. Injection of 1.0 mL of sample in programmed temperature vaporization (PTV) mode at 200°C. The mass spectrometer operated with ion source and interface temperatures set at 200°C, a solvent cut time of 0.05 min, a scan speed of 1111 *amu* min^−1^, and a scan range from *m/z* 42 to 250.

## 3 Results and Discussion

### 3.1 Soret Band Spectral Changes

**FIGURE 2:**
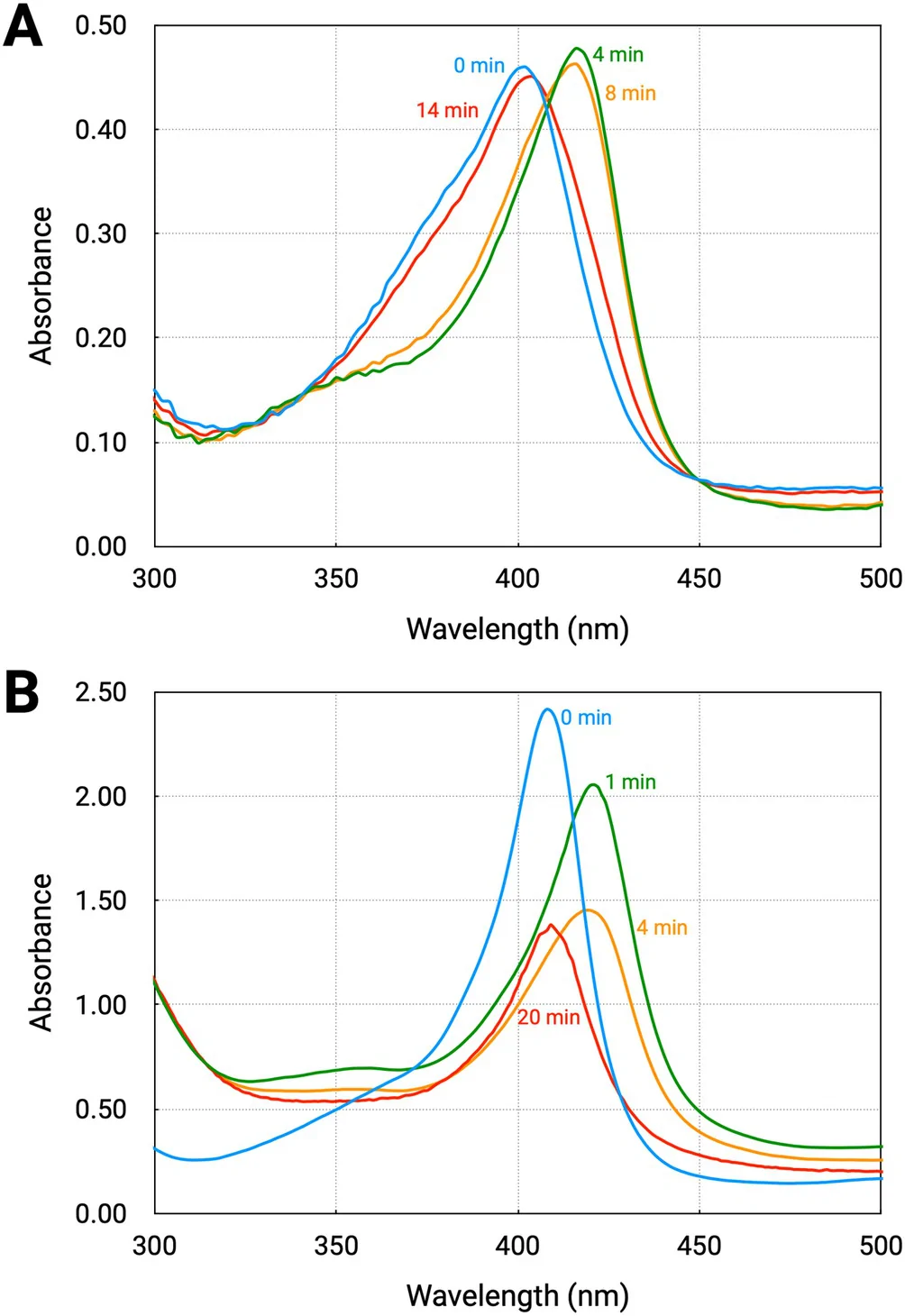
Spectral changes as a function of time for reaction mixtures containing either (A) 10 μM HRP or (B) 10 μM Mb. Spectra were recorded every 2 min. Experimental conditions: 100 μM H_2_O_2_, 20 mM IBAL in 100 mM phosphate buffer, pH 7.4 containing 0.10 mM DTPA.

Figure 2 depicts the time course of the Soret band near 403 nm of HRP during catalysis in comparison with those of ferriMb—Fe^3+^, i.e., intermediates Compound I—Fe^4+^ = O^•+^ and Compound II—Fe^4+^ = O. Reportedly, the absorbance coefficients for HRP are ε_403 nm_ = 1.02 × 10^5^ M^−1^ cm^−1^ for the native enzyme; ε_400 nm_ = 5.38 × 10^4^ M^−1^ cm^−1^ for HRP‐I; and ε_420 nm_ = 1.05 × 10^5^ M^−1^ cm^−1^ for HRP‐II [39]. Expectedly, the maximum absorption of the Soret band in the presence of IBAL was shifted from 400 nm for the native forms, approximately 403 nm for HRP‐I and 420 nm for HRP‐II, with slight wavelength variations observed for Mb [39, 40]. The spectral changes of the native forms of both proteins showed a rapid decay at 403 nm followed by a steady increase at 420 nm in the presence of IBAL to HRP and Mb.

Possibly, the conversion of both hemoproteins into Compound III (•Fe^3+^–O–O–H), from which heme destruction may occur, leads to an enzyme “suicide inactivation” [41]. The spectra traced for Mb at 410 nm (Figure 2B) may indicate extensive protein inactivation accompanied by consequent loss of its peroxidase activity. The observed λ_max_ value is in accordance with that reported for HRP‐III (λ_max_ = 416 nm) [39].

In summary, these results corroborate with HRP undergoing the classical peroxidase catalytic recycling, which is confirmed by a clear isosbestic point at about 411 nm. In contrast, ferriMb seems to initiate the reaction, characterized by the interconversion between the native form, Compound I, and Compound II, but is rapidly inactivated, possibly through the formation of Compound III, thus characterized here as a “pseudoperoxidase.”

### 3.2 Product Analysis

Expectedly, acetone was detected in the ferriMb, H_2_O_2_ and IBAL final reaction mixture by GC–MS (Figure S1), thereby providing further evidence that the protein acts as a pseudoperoxidase (Figure 2B) by comparison with the mechanism previously established for the HRP system [6, 8, 42]. Although the GC–MS spectra exhibited ions at *m/z* 45, which are consistent with formate species, the present study was specifically designed to detect acetone, and no authentic formate standard or dedicated analytical method was employed to confirm its identity.

### 3.3 Oxygen Uptake and CL Studies

**FIGURE 3:**
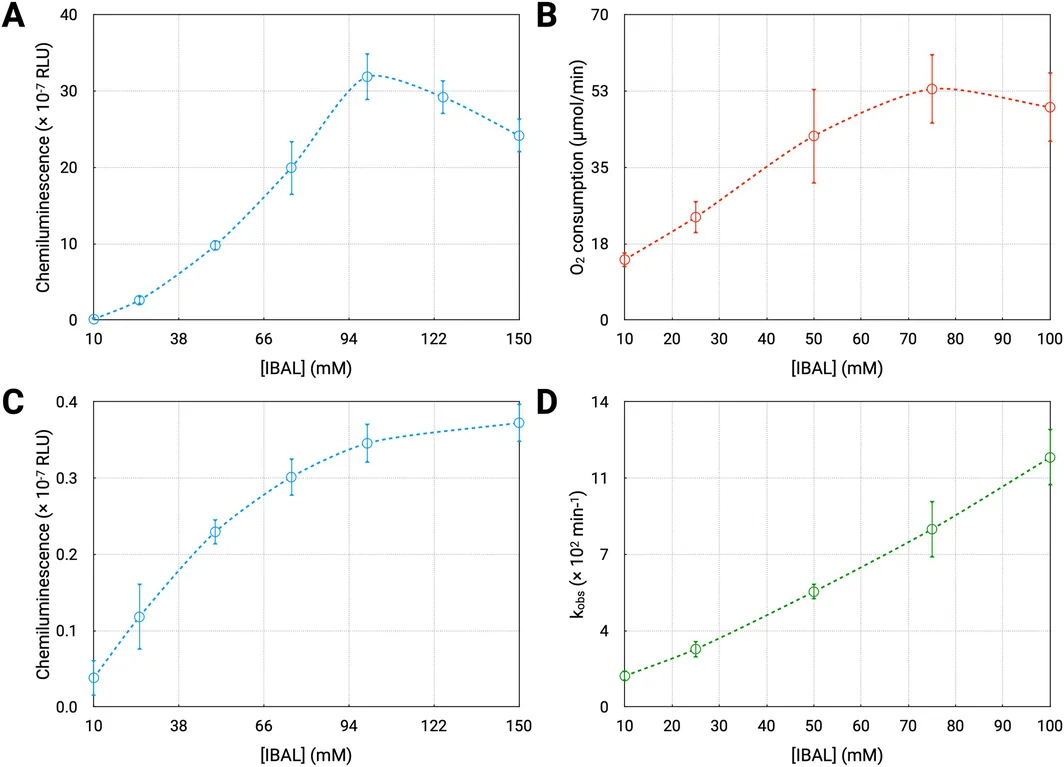
CL intensity (A) and O_2_ consumption (B) by the HRP system, and CL intensity (C), as well as the observed rate constant (k_obs_) of oxygen uptake (D) in ferriMb containing system, plotted as a function of IBAL concentration (0–100 mM). Experimental conditions: 100 μM H_2_O_2_, in 100 mM phosphate buffer, pH 7.4, DTPA 0.10 mM.

Oxygen consumption assays displayed O_2_ depletion at a much shorter time in the presence of HRP than in ferriMb at the same concentration. Moreover, the linear decay for HRP suggests a zero‐order reaction in oxygen concentration. In contrast, ferriMb decay is consistent with first‐order kinetics in oxygen (Figure S2) as previously also reported for indole‐3‐acetic acid oxidation catalyzed by HRP [43]. In comparison with the CL curves, those of oxygen uptake rate for the system containing HRP (Figure 3A,B) and k_obs_ for the system containing ferriMb (Figure 3C,D) as a function of IBAL exhibit very similar pattern, thus supporting the proposed mechanism for both systems.

The CL emission intensity by the system containing HRP is approximately 100‐fold higher than that observed with ferriMb. This difference reflects the fact that HRP has been evolutionarily to function as a peroxidase in plants, whereas Mb primarily serves as a molecular oxygen storage in mammals [17, 44]. In addition, we show here that at higher protein concentrations, Mb can act likewise HRP. Thus, it is possible that Mb from muscles could trigger the generation of triplet acetone from an aldehyde as substrate, accompanied by light emission and cause radical damage to other biomolecules. Unexpected was the fact that in higher protein concentrations, triplet acetone generation resulted in lower intensity of light emission (Figure S3). It is conceivable that the excited product may have been quenched by the HRP and ferriMb proteins themselves, leading to deactivated protein during the reaction (Figure S3). Importantly in this regard, the CL of the created triplet acetone can be partially absorbed by the hemoproteins, whose Soret band maximum at 400 nm overlaps with the triplet acetone emission maximum at 430 nm [6]. Or either, free Mb might abstract hydrogen atoms from the protein and other hydrogen biodonors. The apparent discrepancy of our data with those of Ganini et al. [12], in which the CL intensity of excited methylglyoxal and diacetyl increased with ferriMb concentration, can be attributed to the emission wavelengths of the triplet α‐dicarbonyl products. These excited species emit phosphorescence spanning from 500 to 600 nm region, where absorption by hemoproteins is minimal, and therefore interfere much less than triplet acetone.

### 3.4 Buffer and pH Effect

**FIGURE 4:**
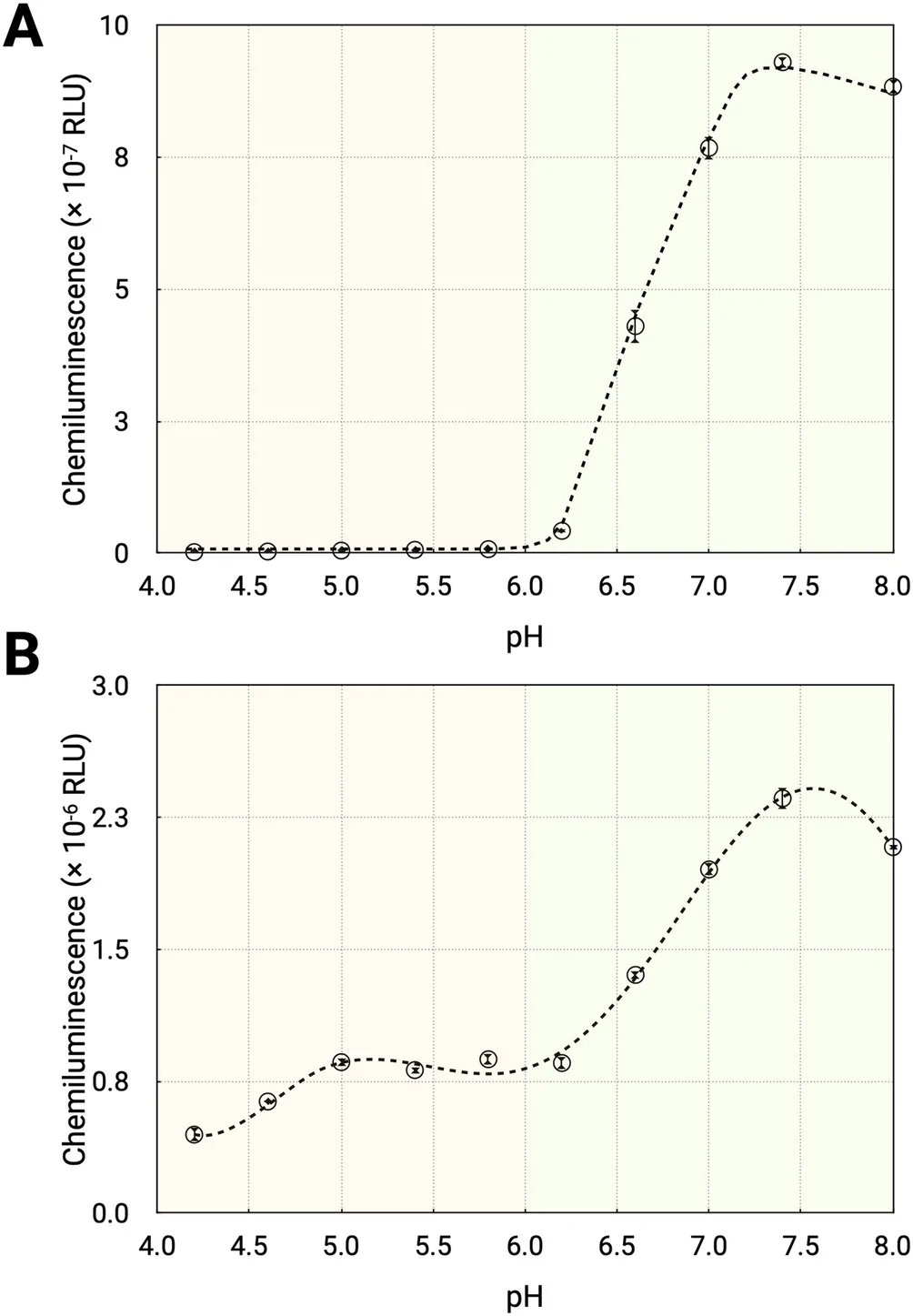
Chemiluminescence as a function of pH: 4.2–5.8 for acetate (pale orange) and 6.2–8.0 for phosphate buffer (pale green), with 10 μM HRP (A) and 10 μM ferriMb (B). Experimental conditions: 100 μM H_2_O_2_, 100 mM IBAL, 100 mM acetate, 100 mM phosphate, and 0.10 mM DTPA.

The CL as a function of pH in phosphate buffer exhibits a very similar profile for both HRP and ferriMb systems, the optimum pH for both systems lying around 7.4 (Figure 4A,B). It is expected for the fact that the dibasic phosphate anion (pK_a_ = 7.20) has been previously reported to catalyze the conversion of IBAL to its enolic form, whose oxidation to the enolyl radical initiates the reaction leading to triplet [6, 8] (Figures 1 and 4). Accordingly, the CL intensity increases with phosphate concentration, reaching a plateau at concentrations above 300 mM (Figure S4) and is much higher than that observed in HEPES or Tris buffer (Figure S5).

### 3.5 Quenching by Sorbates

**FIGURE 5:**
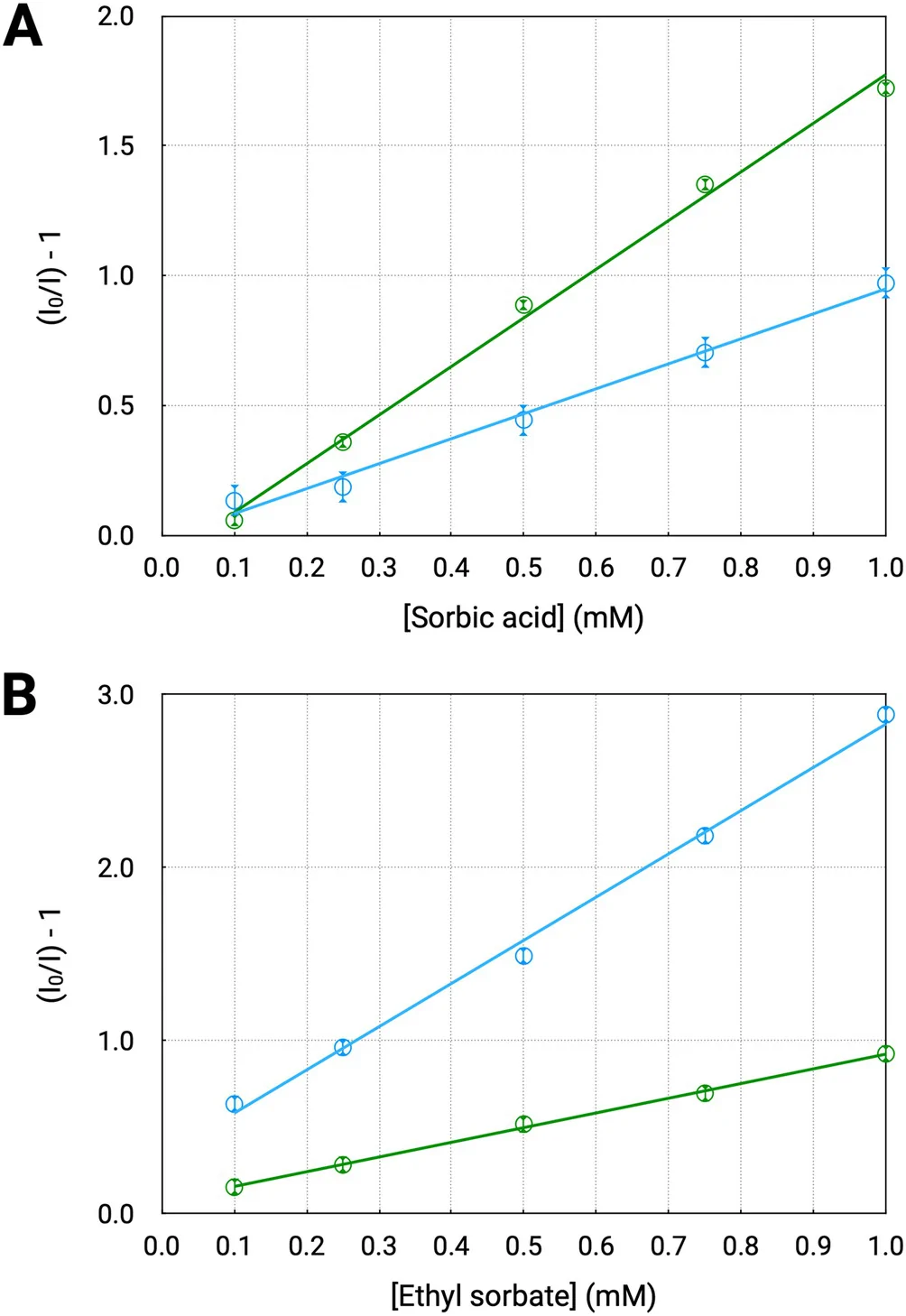
Stern–Volmer plots with sorbic acid (0.10–1.0 mM) as quencher for 10 μM HRP (blue) and 10 μM ferriMb (green) (A) and using ethyl sorbate (0.10–1.0 mM) as quencher for 10 μM HRP (blue) and 10 μM Mb (green) (B). Experimental conditions: 100 μM H_2_O_2_, 100 mM IBAL, in 100 mM phosphate buffer, pH 7.4, 0.10 mM DTPA.

That the emitting excited species generated by the ferriMb system can be attributed to triplet excited acetone is corroborated through Stern–Volmer plots performed with the conjugated dienes quenchers: sorbic acid and ethyl sorbate (Figure 5A,B). Several conjugated dienes have been characterized as efficient quenchers of triplet species in both aqueous and organic solvents [45, 46, 47]. Table 1 shows the corresponding bimolecular quenching rate constants, obtained from the slopes of the resulting plots (K_SV_) and assuming triplet acetone lifetime τ in air‐equilibrated aqueous medium as 1.2 μs (Equation 2) [48].

**TABLE 1:** Stern–Volmer (K_SV_) and quenching rate constants (k_q_) for enzymatically generated triplet acetone challenged with sorbic acid and ethyl sorbate.

Quencher	HRP		ferriMb	
	K_SV_ (× 10^−3^ M^−1^)	k_q_ (× 10^−9^ M^−1^ s^−1^)	K_SV_ (× 10^−3^ M^−1^)	k_q_ (× 10^−9^ M^−1^ s^−1^)
Sorbic acid	0.96 ± 0.03	0.80 ± 0.02	1.9 ± 0.03	1.6 ± 0.02
Ethyl sorbate	2.5 ± 0.2	2.1 ± 0.2	0.85 ± 0.03	0.71 ± 0.03

The kinetic constants indicate collisional quenching processes in water assuming k_diff_ to be roughly 5 × 10^9^ M^−1^ s^−1^ as expected for 1,3‐dienes and the lifetime of triplet acetone in water as close to 1.2 μs [47, 48]. In turn, upon addition of sorbic acid as quencher, k_q_ (Mb) is about twofold higher than that of k_q_ (HRP). In contrast, when ethyl sorbate is used, the pattern is reversed, with k_q_ (HRP) being three times greater than k_q_ (Mb). Considering that (i) HRP is a heavier protein than Mb, i.e., 40 and 17 kDa, respectively; and (ii) the corresponding heme group is buried in HRP whereas more water‐exposed in Mb [49], it is reasonable to assume that the triplet acetone generated by Mb is more susceptible to quenching by sorbate anion than by ethyl sorbate, while the hydrophobic microenvironment of HRP favors ethyl sorbate quenching.

### 3.6 Energy Transfer to DBAS

The dibromo‐sulfonic salt DBAS was employed to further characterize the electronically excited triplet state of acetone produced by the Mb. Addition of DBAS to the ferriMb/H_2_O_2_/IBAL reaction mixture resulted in roughly 1.5‐fold CL enhancement (Figure S6) [48, 50]. Additionally, the DBAS emission was suppressed upon addition of sorbate derivatives, thus confirming the involvement of excited triplet states (Figure S6). Sorbic acid displayed a higher quenching efficiency than ethyl sorbate, which agrees with the Stern–Volmer analysis. Together, the CL and quenching studies support the conclusion of triplet acetone formation by ferriMb‐mediated IBAL oxidation.

### 3.7 SDS‐PAGE Studies of FerriMb

**FIGURE 6:**
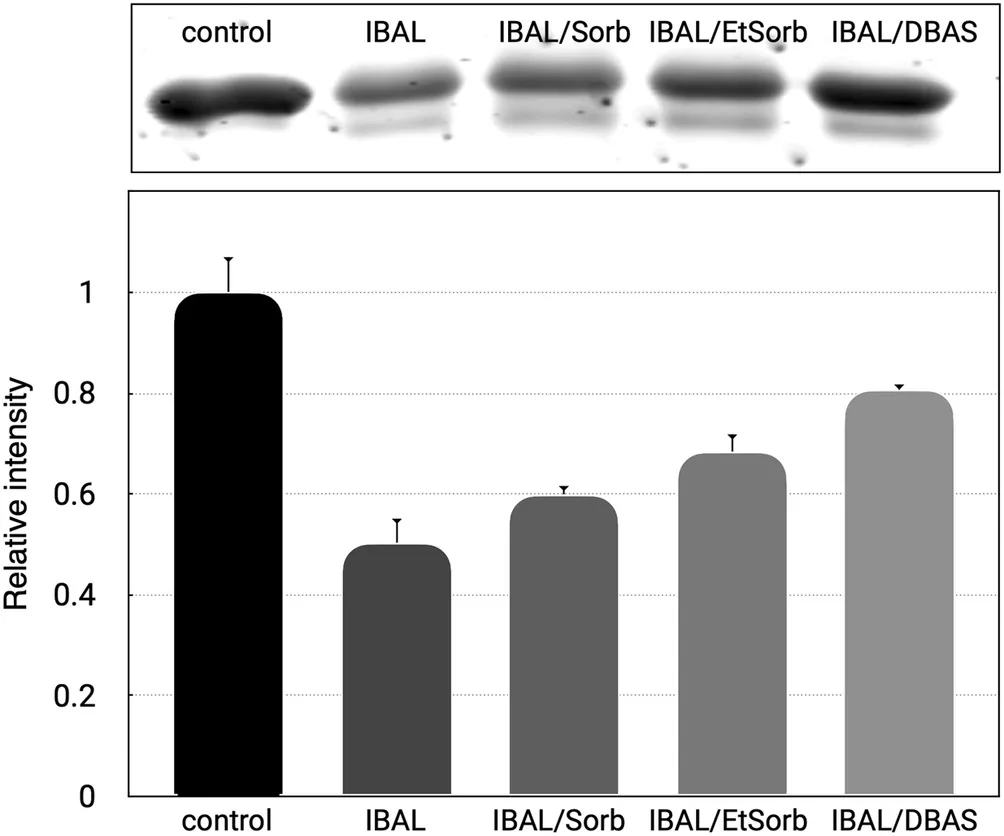
Densitometric analysis of gel bands using ImageJ from a 15% SDS–PAGE of ferriMb (1.0 mg mL^−1^ or 60 μM) incubated for 24 h at 37°C in the control system (1.0 mg mL^−1^ or 60 μM ferriMb plus 100 μM H_2_O_2_), in the absence or presence of 100 mM IBAL, 1.0 mM sorbic acid, 1.0 mM ethyl sorbate, and 5 μM DBAS.

Previous studies reported that triplet acetone can abstract hydrogen from the carbohydrate moiety of the HRP (18%), leading to oxidative damage [51]. In agreement, *iso*‐propanol and the radical dimer pinacol have been identified in the final reaction mixtures [52]. Additional protein damage may also arise from the formation of a Schiff adduct with IBAL [53, 54]. Mb lacks a carbohydrate moiety; nevertheless IBAL was also observed to induce damage to ferriMb (Figure 6). In addition to possible acetone‐Schiff adduct formation with Mb, one cannot discard triplet consumption of excited acetone by the Mb[Tyr^103^] residue, leading to the protein modification [55]. Accordingly, EPR and ESI‐MS spin trapping studies with 5,5‐dimethyl‐1‐pyrroline *N*‐oxide (DMPO) of Mb‐treated H_2_O_2,_ followed by immuno‐spin trapping analysis, revealed the presence of the expected DMPO protein adduct with tyrosyl radical [56, 57, 58]. Structural damage to Mb[Tyr^103^] and Cys^110^ residues by peroxynitrite and H_2_O_2_ is consistent with loss of protein structure and is presumably linked to biological functions associated with proteolysis [59, 60, 61].

Similarly to the HRP‐containing system, addition of the sorbate anion, ethyl sorbate, and DBAS to ferriMb was found to protect the protein structure against induced damage, preserving up to 80% of the protein bands (Figure 6). Additionally, peroxidation of other aldehydes derived from triglycerides and Schiff bases peroxidation may also generate triplet carbonyls and undergo Russel reaction yielding singlet oxygen [62, 63]. Consistent with these observations, the induction of structural damage to Mb by triplet carbonyl species suggests their involvement in protein dysfunction under conditions of carbonyl stress [64]. In this context, it is conceivable that ferriMb does not act strictly as a catalyst, but rather as initiator of the radical chain oxidation of IBAL by oxygen.

Figure 6 attests that reactive triplet acetone produced by the ferriMb/H_2_O_2_ system can accomplish structural damage to the protein. This is reinforced by the band shielding effect upon previous treatment with DBAS as a probe and sorbates as quenchers. Therefore, Mb emerges here with a pseudoperoxidase activity in oxidative and carbonyl stress environments in addition to its well‐established role in oxygen storage in muscles. Together, the observed Mb damage is likely to be importantly caused by direct radical interactions of reactive excited species with specific amino acid residues, such as Tyr^103^ [55] and probably Cys^110^ as well [59].

**FIGURE 7:**
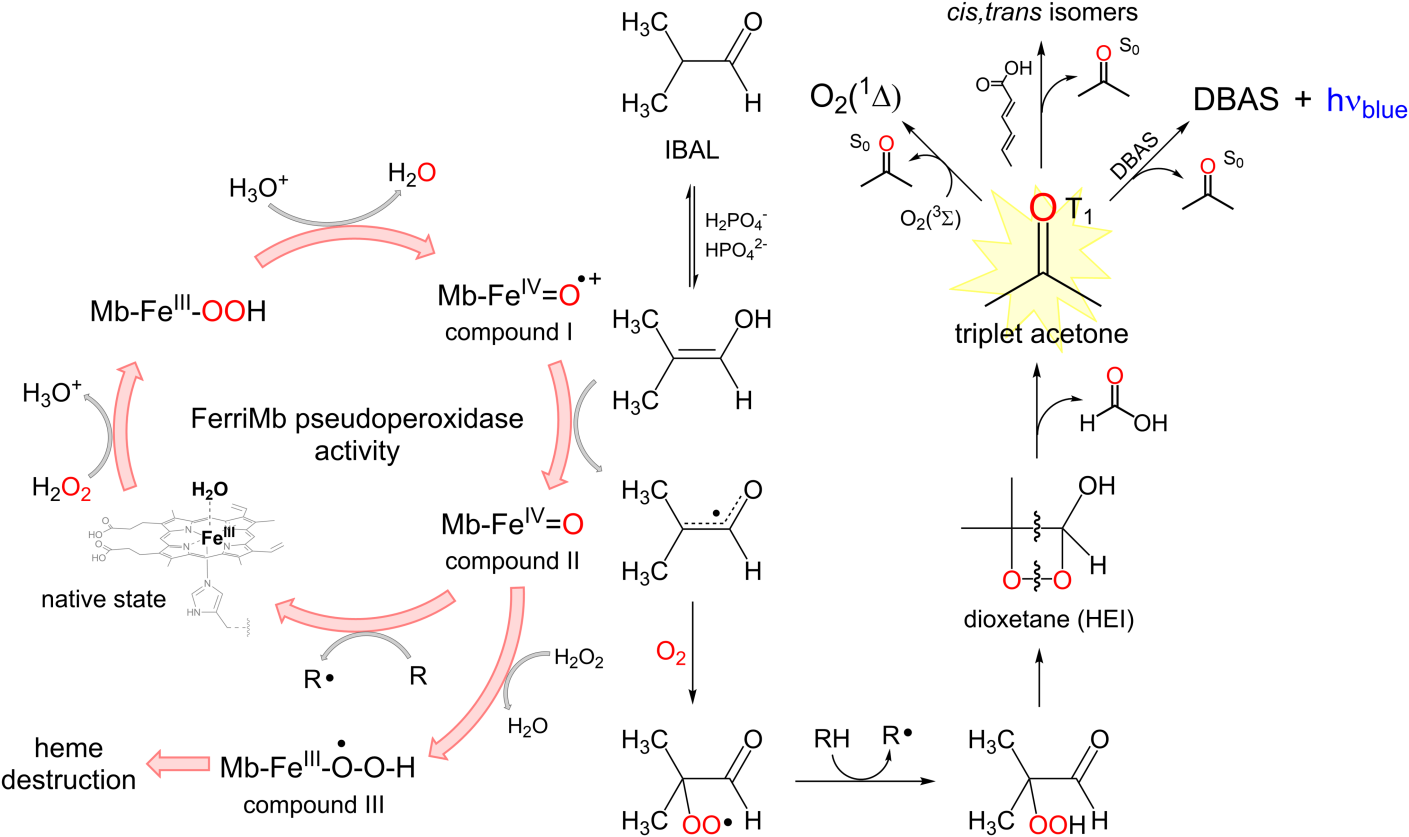
Triplet acetone generation by the aerobic oxidation of IBAL [6, 8] catalyzed by ferriMb pseudoperoxidase activity, illustrating the classical catalytic cycle of peroxidase enzymes as well as possible fates of triplet acetone. Among them, we mention (i) generation of singlet oxygen through triplet‐triplet energy transfer to molecular oxygen [65]; (ii) quenching by sorbic acid, a conjugated *trans, trans‐*diene, leading to the formation of *cis, trans* photoproducts; (iii) excitation of 9, 10‐dibromoanthracene‐2‐sulfonate (DBAS), with consequent blue light emission; and (iv) the potential protein “suicide inactivation”, culminating in heme group degradation via Compound III [41].

Finally, the mechanistic insights reported here may be relevant under pathological conditions with increased extracellular Mb levels, such as rhabdomyolysis, aneurysm rupture and rhabdomyosarcoma [29, 31, 32]. Our data suggest the possible involvement of triplet carbonyl species together with radicals and other reactive oxygen species in Mb‐induced oxidative harmful mechanisms. The protective effect provided by sorbate derivatives like sorbate esters and designed peptides could potentially harness clinical strategies to mitigate Mb lesions caused by excited‐state species under carbonyl stress conditions. The findings of the present work are graphically summarized by Figure 7.

## 4 Final Remarks

This work raises the possibility of heme‐containing proteins to promote the aerobic oxidation of aldehyde metabolites possessing an activated vicinal group, as is the case of a methyl substitution in IBAL, yielding triplet acetone. Electronically photo‐ or chemiexcited products to the triplet state are endowed with the property to trigger chemical damage to protein, other biomolecules, and cell structures [64]. Herein, we report structural and potentially functional damage to Mb by IBAL‐generated triplet acetone. Based on the well‐established ability of conjugated dienes to quench excited triplet states, the efficiency of sorbic acid and its esters in deactivating triplet carbonyl species in this system was evaluated. Noteworthy, peroxidation of other aldehydes derived from triglycerides and Schiff bases formed through Maillard reactions may also generate triplet carbonyls and undergo Russel reaction yielding singlet oxygen [62, 63].

Our data suggest that 1,3‐diene quenchers with increased permeability may have pharmacological potential in preventing the onset of tissue damage linked to rhabdomyolysis, SAH, myosarcomas and other disorders associated with emergence of circulating extracellular Mb.

## Author Contributions

**Thiago M. V. Gomes:** formal analysis, visualization, writing – original draft, writing – review and editing, conceptualization, data curation, methodology, investigation, validation, software. **Cassius V. Stevani:** formal analysis, visualization, writing – original draft, writing – review and editing, funding acquisition. **Kostas Pantopoulos:** writing – review and editing. **Luiz D. Ramos:** conceptualization, data curation, visualization, writing – original draft, methodology, supervision, project administration, writing – review and editing, validation, funding acquisition, resources. **Etelvino J. H. Bechara:** data curation, formal analysis, visualization, writing – original draft, investigation, supervision, project administration, writing – review and editing, validation, funding acquisition, resources, conceptualization, methodology.

## Funding

This work was supported by the Fundação de Amparo à Pesquisa do Estado de São Paulo (FAPESP), project numbers: 2022/03864‐5 (T.M.V.G.); 2025/25849‐6 (T.M.V.G.); 2017/22501‐2 (E.J.H.B., C.V.S.); 2024/15255‐9 (C.V.S.); 2019/24515‐6 (L.D.R.); 2023/04528‐1 (L.D.R.). It was also supported by the Brazilian National Council for Scientific and Technological Development (CNPq), project numbers: 303393/2024‐6 (C.V.S.); 306460/2016‐5 (E.J.H.B.). Additionally, it was supported by the Coordination for the Improvement of Higher Education Personnel‐Brazil (CAPES)‐Finance Code 001.

## Conflicts of Interest

The authors declare no conflicts of interest.

## Supporting information


**Figure S1:** GC–MS chromatograms of samples containing (A) 20 mM IBAL, (B) 20 mM IBAL + 10 mM acetone, and (C) 500 μM ferriMb + 5 mM H_2_O_2_ + 100 mM IBAL after incubation for 24 h at 25°C and 900 rpm under an O_2_ atmosphere. In all chromatograms, the average mass spectrum between retention times of 1.3 and 2.0 min is highlighted, showing the characteristic peaks of IBAL (72 *m/z*) and acetone (58 *m/z*).
**Figure S2:** Molecular oxygen concentration as a function of time at different IBAL concentrations (0–100 mM) for systems containing (A) 10 μM HRP or (B) 10 μM Mb. Experimental conditions: [H_2_O_2_] = 100 μM in 100 mM phosphate buffer (pH 7.4) containing 0.1 mM DTPA.
**Figure S3:** Integrated CL area as a function of (A) HRP concentration (5–50 μM) or (B) ferriMb concentration (5–50 μM). Experimental conditions: [H_2_O_2_] = 50–500 μM, [IBAL] = 100 mM, in 100 mM phosphate buffer (pH 7.4) containing 0.1 mM DTPA.
**Figure S4:** Integrated CL area as a function of phosphate concentration (50–500 mM), for (A) 10 μM HRP and (B) 10 μM ferriMb. Experimental conditions: [H_2_O_2_] = 100 μM, [IBAL] = 100 mM, pH 7.4, and 0.10 mM DTPA.
**Figure S5:** Integrated CL area as a function of pH in phosphate, Tris, and HEPES buffers. Experimental conditions: [ferriMb] = 10 μM, [H_2_O_2_] = 100 μM, [IBAL] = 100 mM, and [DTPA] = 0.10 mM.
**Figure S6:** Integrated CL area for the control system (10 μM ferriMb + 100 μM H_2_O_2_ + 100 mM IBAL) in the absence or presence of 5 μM DBAS, 1.0 mM sorbic acid, and 1.0 mM ethyl sorbate, in 100 mM phosphate buffer (pH 7.4) containing 0.10 mM DTPA.

## Data Availability

The data that support the findings of this study are available from the corresponding author upon reasonable request.
