# Supplementary material for: Triplet Acetone Generation by Pseudoperoxidase Activity of Myoglobin: Structural Damage and Quenching
Source: Luminescence. 2026 Sep 9;41(9):e70618. doi: 10.1002/bio.70618 (PMC13556174; doi:10.1002/bio.70618)
Supplement: Supplementary file 1 [file BIO-41-e70618-s001.docx]

Supplementary Information

**Figure S1.** GC-MS chromatograms of samples containing (A) 20 mM IBAL, (B) 20 mM IBAL + 10 mM acetone, and (C) 500 μM ferriMb + 5 mM H_2_O_2_ + 100 mM IBAL after incubation for 24 h at 25 °C and 900 rpm under an O₂ atmosphere. In all chromatograms, the average mass spectrum between retention times of 1.3 and 2.0 min is highlighted, showing the characteristic peaks of IBAL (72 *m/z*) and acetone (58 *m/z*).

**Figure S2.** Molecular oxygen concentration as a function of time at different IBAL concentrations (0 – 100 mM) for systems containing (A) 10 µM HRP or (B) 10 µM Mb. Experimental conditions: [H_2_O_2_] = 100 µM in 100 mM phosphate buffer (pH 7.4) containing 0.1 mM DTPA.

**Figure S3.** Integrated CL area as a function of (A) HRP concentration (5 – 50 µM) or (B) ferriMb concentration (5 – 50 µM). Experimental conditions: [H_2_O_2_] = 50 – 500 µM, [IBAL] = 100 mM, in 100 mM phosphate buffer (pH 7.4) containing 0.1 mM DTPA.

**Figure S4.** Integrated CL area as a function of phosphate concentration (50 – 500 mM), for (A) 10 µM HRP and (B) 10 µM ferriMb. Experimental conditions: [H_2_O_2_] = 100 µM, [IBAL] = 100 mM, pH 7.4, and 0.10 mM DTPA.

**Figure S5.** Integrated CL area as a function of pH in phosphate, Tris, and HEPES buffers. Experimental conditions: [ferriMb] = 10 µM, [H_2_O_2_] = 100 µM, [IBAL] = 100 mM, and [DTPA] = 0.10 mM.

**Figure S6.** Integrated CL area for the control system (10 µM ferriMb + 100 µM H_2_O_2_ + 100 mM IBAL) in the absence or presence of 5 µM DBAS, 1.0 mM sorbic acid, and 1.0 mM ethyl sorbate, in 100 mM phosphate buffer (pH 7.4) containing 0.10 mM DTPA.
